# A Solid-State Pathway towards the Tunable Carboxylation of Cellulosic Fabrics: Controlling the Surface’s Acidity

**DOI:** 10.3390/membranes11070514

**Published:** 2021-07-08

**Authors:** Eugenio H. Otal, Manuela L. Kim, Juan P. Hinestroza, Mutsumi Kimura

**Affiliations:** 1 Department of Chemistry and Materials, Faculty of Textile Science and Technology, Shinshu University, Ueda Campus, Ueda, Nagano 386-8567, Japan; manuela_kim@shinshu-u.ac.jp; 2 Department of Fiber Science and Apparel Design, Cornell University, Ithaca, NY 14853, USA; jh433@cornell.edu; 3 COI Aqua-Innovation Center, Shinshu University, Ueda Campus, Ueda, Nagano 386-8567, Japan; 4 Research Initiative for Supra-Materials, Shinshu University, Ueda Campus, Ueda, Nagano 386-8567, Japan

**Keywords:** cellulose carboxylation, solid state reaction, surface acidity, cationic dye uptake

## Abstract

We report on a tunable solid-state approach to modify the acidity of cotton substrates using citric, oxalic, and fumaric acids. The first stage of the method involves soaking the cotton swatches in an ethanolic saturated solution of the corresponding acid. After drying, the carboxylation reaction proceeds at high temperature (T > 100 °C) and in solid state. We quantified the effect of temperature and reaction time on the solid-state carboxylation reaction, which allowed us to tune the carboxylation degree and the acidity of the surface. We characterized the modified cotton by performing adsorption isotherms and by determining the kinetics of adsorption of a cationic dye: methylene blue (MB). We found that the MB uptake kinetics varied as a function of the acidic strength of the surface, which is closely related to the strength of the acid used for surface modification. The proposed solid-state cotton carboxylation procedure allows us to achieve sustainable cotton modification, which constitutes a starting point for several applications using cotton as the substrate.

## 1. Introduction

Modifying the surface chemistry of cotton can enhance the interaction of cotton with charged species such as metals ions and cationic dyes. The surface complexation of metals on the surface of fabrics can be used in many applications such as metal uptake from polluted streams [1] as well as heteroepitaxial growth of MOF structures [2,3,4,5], which can improve the performance and selectivity of the filtering performance [6,7]. Carboxylated cellulose also increases dye attachment [8] and can be used as a non-formaldehyde treatment for durable press finishing and anti-wrinkle treatments [9].

The modification of cellulose with carboxyl groups can be achieved with various methods such as using sodium chloroacetate in basic media [10] or by TEMPO oxidation [11], mechanochemical esterification [12], etc. However, these methods have limitations in controlling the rate of the reaction, and sometimes, they require expensive toxic reagents, several reaction steps, or low reaction yield. The introduction of carboxylic groups via a Fischer esterification reaction using acid catalyst and heating is one of the oldest and most common ways to introduce carboxylic groups into cellulose [13,14]. The reversibility of this reaction can be controlled through the elimination of the reaction products (water and esters), hence displacing the equilibrium and increasing its yield. The distillation of byproducts during this reaction is a common practice in industrial processes, but it is only possible when reagents have a boiling point higher than that of water and of the desirable products.

When polycarboxylic acids react with cellulose, one carboxylic group attaches to the –OH groups from the cellulose, normally C_6_. The rest of carboxylic groups remain free or react with an –OH group from another glucose unit, hence enabling the crosslinking of cellulose. By controlling the reaction conditions and by properly choosing the polycarboxylic acids, it is possible to control the number of free carboxy content (FCC) that quantifies the carboxyl groups not involved in ester bonds. The total carboxyl content (TCC) includes the free and the esterified carboxyl groups. The ratio between FCC and TCC quantifies the amount of free carboxyl groups after carboxylation with respect to the total carboxylates available before the reaction [15]. The FCC/TCC ratio also indicates the degree of crosslinking between cellulosic chains and the polycarboxylic acid. When less free carboxylates are available, the degree of crosslinking increases. For a dicarboxylic acid, this ratio varies between 0 (full crosslinking) and ½ (no crosslinking), and in the case of tricarboxylic acids, this ratio goes from 0 to 2/3. Another relevant metric is the degree of substitution (DS), which quantifies the amount of –OH groups in position C_6_ that are esterified.

In this work, we report on the solid-state carboxylation of two types of cotton fabrics using oxalic, fumaric, and citric acid as functionalizing reagents. We also perform optimization studies on reaction time and temperature aimed at obtaining carboxylated cotton fabrics with controllable amounts of free carboxy groups and crosslinking degrees. The as-obtained materials were tested in the absorption of methylene blue (MB), showing the possibility of using these materials in pollutant adsorption applications.

## 2. Materials and Methods

**Figure 1:**
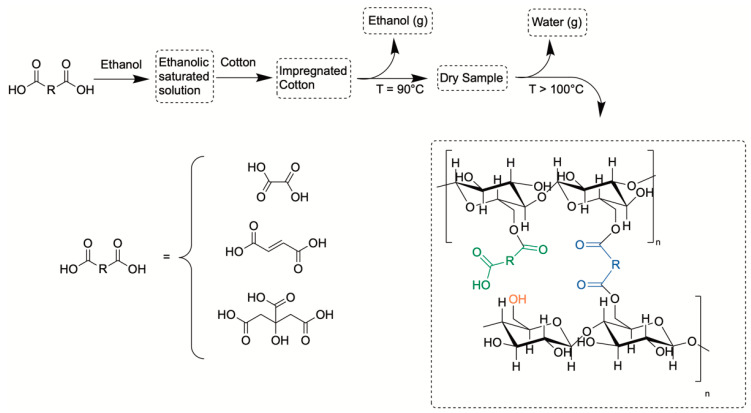
Schema showing the procedure used for cotton modification (**top**). Chemical structure of the polycarboxylic acids used in the proposed method (**bottom**-**left**). Possible resulting products of the proposed method (**bottom**-**right**). The green moieties represent the free carboxy groups (FCC), and the blue ones represent the crosslinked groups. Orange moieties correspond to the –OH group from C_6_.

Oxalic acid (C_2_H_2_O_4_,), fumaric acid (C_4_H_4_O_4_), and citric acid (C_6_H_8_O_7_) (see the chemical structures in Figure 1) were purchased from FUJIFILM Wako Pure Chemical Corporation (Osaka, Japan). Two types of plain-woven 100% cotton fabrics were used, one manufactured in Japan (JP cotton, Kitamura Co., Aichi, Japan) and another manufactured in the USA (US cotton, TIC-400, Testfabrics, Inc., West Pittston, PA, USA). The JP cotton was bleached; had an optical brightener; and had 16 yarns per cm, with the yarns having a dtex_JP_ = 263. The US cotton specimens were not bleached and had 26 yarns per cm, and the yarns had a dtex_US_ = 158.

The cotton samples were washed following a modified report by Schelling et al. [5]. The scouring solution was prepared by dissolving 5 g of NaOH, 1.5 g of Triton X, and 0.75 g of citric acid in 500 mL of DI water. The cotton samples were cut to the size 1 inch × 1 inch and immersed in the scouring solution at 100 °C for 1 h. The samples were rinsed several times with DI water and then dried at 80 °C. Ethanolic saturated solutions of oxalic, fumaric, and citric acid were prepared by adding the solid to the solvent and by stirring overnight until a solid acid remained. The cotton swatches were dipped into 100 mL of saturated ethanolic solutions at room temperature and dried at 90 °C for 5 min. The influence of the solid-state reaction time at 110 °C was studied by removing the samples after 15, 30, 45, 60, 90, 120, and 180 min. Temperature-dependent experiments were performed for 45 min in the range of 110 °C to 170 °C for citric acid and 110 °C to 200 °C for fumaric acid. The oxalic acid experiments were performed only at 110 °C. Further washing of the samples was performed twice in DI water using an orbital shaker for 2 h and then twice in ethanol using an ultrasonic bath for 20 min to remove the unreacted acids.

The FTIR experiments were performed in a Bruker Alpha II (Bruker, Billerica, MA, USA). The total carboxyl content (TCC) of modified cotton samples was determined via alkaline hydrolysis and conductimetric back titration. All NaOH and HCl solutions were standardized using potassium hydrogen phthalate and Na_2_CO_3_ as primary solid standards.

A slightly modified procedure from da Silva Perez et al. was used for carboxylate group quantification [15]. Approximately 100 mg of each modified cotton sample was added to 10 mL of NaOH 0.01 M and 20 mL MilliQ water and allowed to stabilize for 30 min. The samples were then titrated with HCl 0.01 M. The TCC was calculated according to the following:

(1) $$\mathrm{TCC}=\frac{C_{\mathrm{HCl}}*\left(V_{\mathrm{blank}}-V_{\mathrm{sample}}\right)}{m_{\mathrm{sample}}}$$

where C_HCl_ is the concentration (in mol L^−1^) of the HCl solution; V_blank_ and V_sample_ are the volumes of HCl used to titrate the non-treated and treated samples, respectively; and m_sample_ is the mass of the dry sample.

For free carboxyl content (FCC) quantification, approximately 100 mg of modified cotton was added to 10 mL of NaCl 0.01 M. The suspension was stirred for 1 h and titrated with 0.01 M of NaOH. The FCC was calculated according to the following:

(2) $$\mathrm{FCC}=\frac{C_{\mathrm{NaOH}}\times V_{\mathrm{NaOH}}}{m_{\mathrm{sample}}}$$

where C_NaOH_ is the concentration (mol L^−1^) of the NaOH solution, V_NaOH_ is the volume of NaOH used to titrate the treated samples, and m_sample_ is the mass of the dry sample. The degree of substitution (DS) was calculated according to the following:

(3) $$\mathrm{DS}=\frac{162.14{\;g\;\mathrm{mol}}^{-1}\times C_{\mathrm{HCl}}\times \left(V_{\mathrm{blank}}-V_{\mathrm{sample}}\right)\times \left(1-\frac{\mathrm{FCC}}{\mathrm{TCC}}\right)}{m_{\mathrm{sample}}-C_{\mathrm{HCl}}\times \left(V_{\mathrm{blank}}-V_{\mathrm{sample}}\right)\times \left({\mathrm{Mr}}_{\mathrm{acid}}-18.02{\;g\;\mathrm{mol}}^{-1}\right)}$$

where 162.14 g mol^−1^ is the average molecular weight of glucose in the cellulose and Mr_acid_ is the molecular weight of the acid used for the cotton modification: oxalic acid (C_2_H_2_O_4_, 90.03 g/mol), fumaric acid (C_4_H_4_O_4_, 116.07 g/mol), and citric acid (C_6_H_8_O_7_, 192.12 g/mol).

Methylene blue (MB) uptake and MB adsorption experiments were performed using batch measurements and aqueous MB solutions. A calibration curve using standard solutions of MB with concentrations ranging from 1–50 mg/L was used. MB quantification was performed by measuring absorbance at 663 nm in a UV spectrophotometer. For pH optimization experiments, a 10 mg/L solution of MB was prepared by proper dilution from a 1000 mg/L stock solution using DI water. The pH values of the MB solution were adjusted between 2 and 10 using proper amounts of HCl 0.01 M or NaOH 0.01 M. Small amounts of modified cotton (50 mg approximately) were placed in 15 mL falcon tubes, and 10 mL of the pH adjusted MB solution was added. The samples were placed on a rotary plate, left overnight to reach equilibrium at room temperature, and kept in the dark. For the adsorption isotherm experiments, approximately 10 mg of modified cotton samples and 10 mL of MB solutions with concentrations ranging from 5–90 mg/L at natural pH value (7–8) were placed in 15 mL conical tubes and left overnight under constant agitation in contact with the samples at room temperature and kept in the dark. Absorbance at 663 nm was measured using a UV-Vis spectrophotometer at 663 nm, and the resultant solutions were properly diluted when needed for the measurements. The amount of adsorbed dye was calculated according to Hameed et al. [16]:

(4) $$q_{\mathrm{eq}}=\frac{\left(C_{i}-C_{\mathrm{eq}}\right)}{m}V_{\mathrm{MB}}$$

where q_eq_ is the amount of adsorbed MB (in mg) per gram of cotton sample, Ci is the initial concentration of MB, C_eq_ is the equilibrium concentration of MB, V is the volume of MB solution used, and m is the cotton sample’s weight. Two models were used for isotherms fitting: the linearized Langmuir equation (Equation (5)) and the logarithmic form of Freundlich isotherm (Equation (6)).

(5) $$\frac{C_{\mathrm{eq}}}{q_{\mathrm{eq}}}=\frac{1}{b\times Q_{m}}+\frac{C_{\mathrm{eq}}}{Q_{m}}$$

(6) $$\log q_{\mathrm{eq}}=\log k_{f}+\frac{1}{n}\log C_{\mathrm{ef}}$$

For the adsorption kinetics measurements, 10 mg of the modified cotton samples were placed in a plastic cuvette with a small magnetic stirrer. At time t = t_0_, 2.5 mL of MB 10 mg/L at natural pH value (7–8) was added to the cuvette, and the absorbance at 663 nm was measured as a function of time. Three models were used for kinetic fitting: pseudo first-order kinetics (Equation (7)), pseudo second-order kinetics (Equation (8)), and intra-particle diffusion (Equation (9)) [17]:

(7) $$\log\left(q_{\mathrm{eq}}-q_{t}\right)=\log\left(q_{\mathrm{eq}}-\frac{k_{1}t}{2.303}\right)$$

(8) $$\frac{t}{q_{t}}=\frac{1}{k_{2}q_{\mathrm{eq}}^{2}}+\frac{t}{q_{\mathrm{eq}}}$$

(9) $$q_{t}=k_{\mathrm{id}}t^{0.5}+c\;$$

where q_eq_ is the amount adsorbed per gram of adsorbent, q_t_ is the amount adsorbed per gram of adsorbent at time t, k_1_ (min^−1^) is the pseudo first-order adsorption rate constant, k_2_ (g·mg^−1^ min^−1^) is the pseudo second-order velocity constant, k_id_ (mg·g^−1^ min^−1/2^) is the intra-particle diffusion constant, and C (mg·g^−1^) is the constant related to diffusion resistance.

## 3. Results

### 3.1. Solid-State Carboxylation Procedure

Figure 1 shows the procedure followed for cotton carboxylation using saturated ethanolic solutions of the carboxylic acids, an impregnation step of the cotton fabric, and the drying/solid-state carboxylation step at temperatures higher than 100 °C. Additionally, the possible reactions found during the solid-state carboxylation procedure are depicted in Figure 1, where the green moieties represent mono-esterified moieties, the blue ones represent the crosslinked groups, and the orange ones represent C_6_ –OH groups.

### 3.2. Time of Reaction Experimental Optimization

The optimal experimental conditions for carboxylation are reported in Table 1. First, the time of reaction was optimized, using 110 °C to ensure water elimination. The highest values of the carboxyl groups are obtained in samples treated with oxalic acid for 90 min, and the lowest number is obtained in samples treated with fumaric acid for 45 min.

### 3.3. Temperature of Reaction Experimental Optimization

Experimental temperature optimization was performed at 45 min of reaction time for fumaric (melting point: 287 °C) and citric acid (melting point: 153 °C). The aim was to work at temperatures not far from the melting points of each acid and to thus enable the solid-state reaction while avoiding a molten state. For oxalic acid, the melting point = 102 °C, prevented us from performing this temperature optimization. Table 2 shows the reaction parameters for samples treated with fumaric and citric acid at different temperatures. The samples treated with fumaric acid did not show additional improvement as temperature increased. In the case of the samples treated with citric acid, a small increase in FCC/TCC and DS with the increase in temperature was noted. We also noted that the type of cotton used does not appear to influence the results, even though both cotton samples had different starting points (one was bleached and contained an optical brightener, whereas the other did not).

### 3.4. FTIR

For all specimens, the FTIR spectra show a carboxyl peak at around 1700 cm^−1^. Samples treated with oxalic acid showed a peak at 1723 cm^−1^, samples modified with fumaric acid showed a peak at 1702 cm^−1^ and those treated with citric acid showed a peak at 1714 cm^−1^. The peak intensity increased with reaction time, hence indicating an increase in the amount of carboxyl groups. The FTIR spectra of treated cotton samples with acids are shown in Figures S1–S3.

### 3.5. Surface Acidity

**Figure 2:**
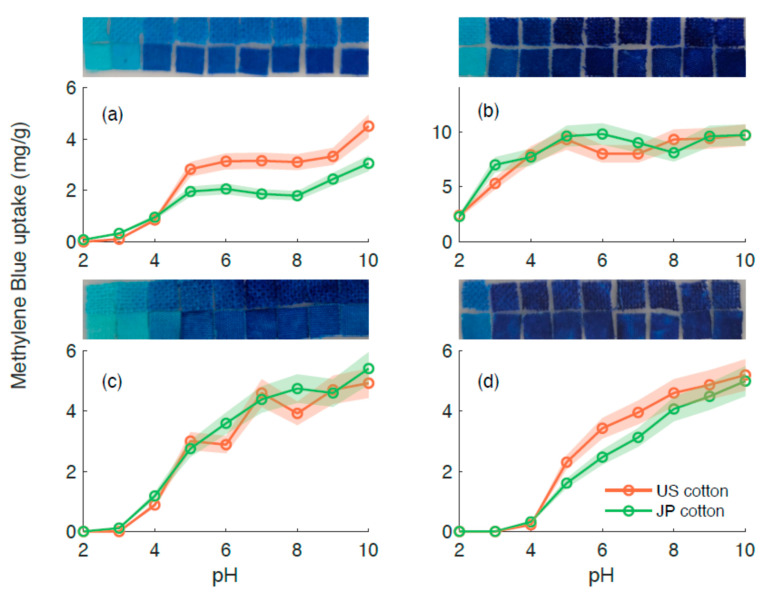
Methylene blue (MB) uptake and resultant color change in carboxylated cotton fabrics as a function of pH. (**a**) Untreated cotton, (**b**) oxalic acid-treated cotton, (**c**) fumaric acid-treated cotton, and (**d**) citric acid-treated cotton. Photographs of the cotton samples aligned with the corresponding pH scale are located at the top of each plot, the top row corresponds to JP cotton, and the bottom row corresponds to US cotton. The shaded areas correspond to confidence intervals.

The incorporation of carboxy groups to the cotton samples increases the acidity of the surface and makes them prone to surface charge changes as a function of pH. To test the surface charge as a function of pH, a cationic dye, methylene blue (MB), was used as a probe (Figure 2) [18]. Samples treated with oxalic acid exhibited MB uptake at pH = 2, indicating that MB uptake onset is below pH = 2. The onset uptake for fumaric acid and citric acid was observed at around pH = 4. Further discussions are presented Section 4.

### 3.6. Dye Adsorption Isotherms

**Figure 3:**
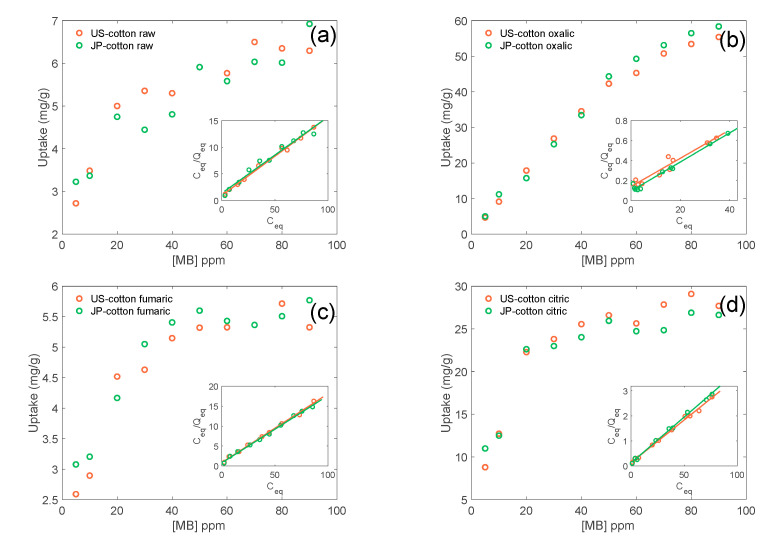
Methylene blue isotherms of (**a**) untreated cotton, (**b**) oxalic acid-treated cotton, (**c**) fumaric acid-treated cotton, and (**d**) citric acid treated cotton. Inset shows the Langmuir linearization of the isotherm.

The optimal pH value for MB adsorption was found to be 7 for fumaric and oxalic acid. In the case of the samples treated with citric acid, the MB uptake appears to monotonically increase with pH and no maximum was noted in the range of our experiments. Isotherms were constructed at pH = 7 and modeled using the most common adsorption isotherms models, Langmuir and Freundlich. The adsorption isotherms have a quantitative agreement with the Langmuir adsorption model (Figure 3), which assumes a monolayer model.

### 3.7. Absorption Kinetics

The uptake kinetics for MB were determined to be of pseudo first-order for oxalic acid and pseudo second-order for fumaric and citric acid. The model definition and fittings are provided in Equations (7) and (8). The carboxyl group titration data are shown in Figures S4–S18. The methylene blue adsorption data are shown in Figures S19–S39.

## 4. Discussion

The proposed method allows us to achieve a tunable cotton carboxylation via two ways: (1) by changing the polycarboxylic acid in the solid-state carboxylation process or (2) by changing the time process.

The increase in time of the solid-state reaction produces an increase in the TCC in all of the cases (Figures S4–S12), asymptotically reaching the maximum TCC value shown in Table 1. On the other hand, the increase in the temperature of the process far above the optimal temperatures (reported in Figures S13–S18) produced a neglectable change or even a decrease in TCC, a yellowing of the cotton fabric, and a decrease in the structural integrity of the fabric due to dehydration [19] and thermal degradation of the cellulose [20,21]. Therefore, the optimal temperatures were chosen (Table 2) for further experiments.

The TCC was compared to that obtained via other methods, as shown in Table 3. Not only was the TCC obtained using the method described in this work higher but also this method allows for better control of the carboxylation degree, avoiding the use of toxic reagents and allowing the unreacted acids to be recycled. Additionally, the proposed method does not use additional catalyzers [21,22], freeze-drying steps [23], etc.

Since oxalic acid is a dicarboxylic acid and citric acid is a tricarboxylic acid, the values of FCC/TCC have different implications. FCC/TCC is defined as follows:

(10) $$\frac{\mathrm{FCC}}{\mathrm{TCC}}=\frac{N_{\mathrm{COOH}\mathrm{reacted}}}{N_{\mathrm{COOH}\mathrm{total}}}$$

For dicarboxylic acids, we have the following cases:

- FCC/TCC = 0 implies that there are no free carboxyl groups and that all of the carboxyl groups form ester bonds.
- 0 > FCC/TCC > 1/2 indicates that there are free carboxyl groups and that ester bonds form.
- FCC/TCC = 1/2 means that there are no ester bonds present, hence representing the maximum number of free carboxyl groups at a given degree of substitution.

In the case of tricarboxylic acids, a more complex situation arises:

- FCC/TCC = 0 implies that there are no free carboxyl groups as they all form ester bonds.
- If 0 > FCC/TCC ≥ 1/3, there is an average of up to one COOH free.
- If 1/3 > FCC/TCC ≥ 2/3, there is an average of up to two COOH free.
- If FCC/TCC = 2/3, there is no crosslinking and the maximum amount of free carboxyl groups is achieved.

**Figure 4:**
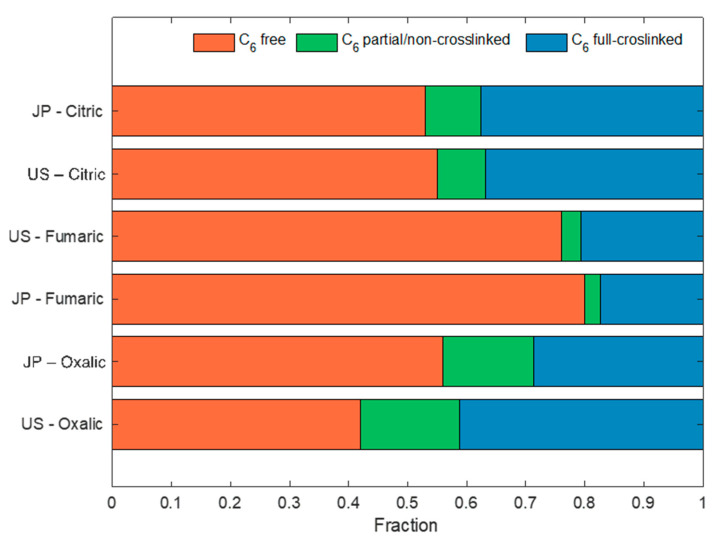
Distribution of C_6_ reaction in different cotton types treated with different polycarboxylic acids under optimized conditions for each acid.

Figure 4 exhibits the percentage of free C_6_; C_6_ bonded to polycarboxylic acid but not crosslinked; and C_6_ bonded to polycarboxylic acid and crosslinked, calculated according to Equations (1) and (2). We assume that the carboxylation occurs mainly in the C_6_ position due to the higher reactivity of the primary –OH with respect to the secondary –OH groups [27]. In the case of citric acid (a tricarboxylic acid), if two carboxylates are esterified, the C_6_ is crosslinked with a free carboxylate, fulfilling the conditions to be defined as crosslinked and non-crosslinked; in this case, we define it as partially crosslinked in Figure 4. The calculated FCC/TCC for citric acid was smaller than 1/3, indicating that, on average, less than one carboxyl group is free and not participating in a crosslinking bond. That is, more than 2 of 3 carboxyl groups participate in a crosslink and are functionalized between 45 and 47% of the C_6_ available on the cotton substrate. Fumaric acid had the lesser degree of functionalization, affecting only between 20 to 24% of C_6_ on the cotton substrate, with around 2–3% of fumarates present as free carboxy groups and with the remaining involved in crosslinking. Oxalic acid showed the best carboxylation performance, affecting 44 to 58% of all C_6_ available. With respect to free carboxyl groups in the oxalate-modified samples, this represents around 15–17% of carboxyl groups. These results indicate that, by changing the type of polycarboxylic acid used, it is possible to tune the number of free carboxylate groups and the degree of crosslinking of cotton.

A mechanism for solid-state esterification has not yet been elucidated [28] and cannot be related to Fischer esterification due to the absence of a solvent and hence carbonyl protonation [29]. The lack of a reaction mechanism makes it difficult to explain the differences in reactivity between the different polycarboxylic acids used. Pantze et al. reported the solid-state esterification of filter paper with different carboxylic acids [30]. They found that 2-ketobutyric acid and 3-hydroxybutiryc acid exhibited good yields with respect to veratric acid, which did not react. Particularly for α-hidroxyacids such as citric acid, a two-step nucleophilic reaction was proposed [31,32]. These results indicate that the esterification reaction is favored by the presence of certain groups, and they agree with our observations (Table 1 and Table 2 and Figure 4). With respect to surface acidity, the free carboxyl group in the oxalic acid-modified samples have a strong inductive effect on the ester carboxylate, showing strong acidity, which is reflected in the partial ionization at pH = 2. The inductive effect of the ester carboxylate in the acidity of the remaining non-crosslinked carboxylic groups of fumaric and citric acid-modified cotton is weaker due to the distance between these moieties (Figure 1). This behavior is also observed in the ${\mathrm{pK}}_{a1}$ values for dicarboxylic acids ((CH_2_)_n_(CO_2_H)), where the stronger inductive effect is observed between the values for oxalic acid (n = 0, ${\mathrm{pK}}_{a1}$ = 1.27) and succinic acid (n = 2, ${\mathrm{pK}}_{a1}$ = 4.21). When the length of the aliphatic chain and distance between the carboxyl groups increase, the ${\mathrm{pK}}_{a1}$ slowly converges to 4.7 [33]. Therefore, at a pH higher than ${\mathrm{pK}}_{a-\mathrm{MB}}$ = 3.1–3.8 [17], the electrostatic interaction between MB and the modified cotton is favored.

At water’s natural pH, cotton’s surface is negatively charged and MB is positively charged, so the first monolayer of MB is expected to strongly interact with the surface. The next layers of MB interact with a surface covered with positively charged MB, making further adsorption unfavorable, in agreement with the Langmuir model. The maximum values for adsorption capacity, Q_0_, for each system are shown in the Supplementary Materials. The highest values of Q_0_ are noted for the samples treated with oxalic acid, and the lowest ones are noted for the samples treated with fumaric acid. For samples treated with fumaric acid, the adsorption capacity is similar to that of untreated cotton. Even though the obtained values for oxalic acid-modified cotton are around half of the adsorption capacity of activated charcoal (160 mg/g) [34] and in the same order of clays (58 mg/g) [18], cotton fabrics have the advantage of being easy to remove without filtration, centrifugation, or another physical separation method, and they can be converted into a sustainable filter to eliminate cationic pollutants from water, e.g., metals and dyes.

For our experiments, we postulate that surface deprotonation is one of the steps involved in MB adsorption. As observed in Figure 2, samples treated with oxalic acid have a plateau in MB adsorption in the range between pH = 5 and pH = 10, confirming that, at pH = 7, the surface of the cotton is fully deprotonated. In the case of samples treated with fumaric and citric acid, there is a monotonically increasing curve, indicating that the surface groups were not fully deprotonated. Taking all of these arguments into account, the pseudo second-order kinetics observed in the fumaric and citric acid-treated samples can be explained using a two-step mechanism: surface deprotonation and dye adsorption. In contrast, in the case of the samples treated with oxalic acid, deprotonation is not part of the adsorption mechanism.

Gürses et al. studied the adsorption of MB onto clays, observing pseudo second-order kinetics [18]. They pointed out that more than one step could be involved in the adsorption process. Clays, alkali, or other metals exchanged on the surface and in the interlayer region of the solid can influence the adsorption process. Bujdak and Komadel studied the interaction of dyes with montmorillonite and reported that dyes were adsorbed via ion exchange [35]. Due to the porous nature of the cotton fibers, the overall adsorption process may be controlled by more than one step as well, e.g., diffusion from the bulk solution, pore diffusion, surface diffusion, pore surface adsorption, or combinations thereof.

All of the experiments were performed in a stirred spectrophotometric cuvette, allowing the mass transfer process from the bulk to the surface of the cotton to be neglected. However, intra-particle diffusion is present, and its influence was explored using a Weber–Morris plot of q_t_ vs. t^1/2^ (Equation (9)). The plots obtained are available in the Supplementary Materials, and they exhibited more than one slope, hence indicating the presence of two absorption steps. The first slope represents the external resistance to mass transfer, and the second slope is an indication of an adsorption stage controlled by intra-particle diffusion [36].

## 5. Conclusions

We presented a simple method for the carboxylation of cellulose that involves soaking cotton fabrics in saturated alcoholic solutions of polycarboxylic acids followed by a thermal treatment. The optimal temperature for maximum FCC was found between 110 and 130 °C. The reaction showed higher FCC/TCC ratios for oxalic acid and citric acid and lower ones for fumaric acid. These discrepancies cannot yet be explained due to the lack of a mechanism for solid-state esterification reactions, but the results are in quantitative agreement with previous observations. The acidity of the surface was found to be proportionally related to the ${\mathrm{pK}}_{a}$s of the acids used, hence showing the influence of the induction effect of near ester groups. At pH = 2, the oxalic acid-treated sample adsorbed a model cationic dye (i.e., methylene blue), while the onset for dye adsorption in the fumaric and citric acid-treated samples was found to be around pH = 4. The fumaric and citric acid-treated samples followed pseudo second-order kinetics, while the oxalic acid-treated samples followed a pseudo first-order model, which can be attributed to a two-step MB uptake mechanism, surface deprotonation, and dye adsorption and to a direct dye adsorption, respectively. The present results show the capability of the modified cotton to be implemented as filters with tunable surface charge as a function of pH, allowing for selective filtering of charged species.

## Figures and Tables

**Table 1:** Optimized times, total carboxyl groups content (TCC), free carboxyl groups (FCC), and FCC/TCC ratios for samples treated with oxalic, fumaric, and citric acid at 110 °C.

	Optimal Time (min)	TCC (mmol/g)	FCC (mmol/g)	FCC/TCC	DS
US—Oxalic	90	4.0	1.1	0.29	0.58
JP—Oxalic	90	3.5	1.2	0.35	0.44
US—Fumaric	45	1.3	0.17	0.13	0.21
JP—Fumaric	45	1.5	0.22	0.15	0.27
US—Citric	60	2.2	0.35	0.16	0.45
JP—Citric	45	2.3	0.35	0.15	0.49

**Table 2:** Optimal temperature, total carboxyl groups content (TCC), free carboxyl groups content to total carboxyl groups content ratio, and degree of substitution determined for samples treated with fumaric and citric acid for 45 min.

	Optimal Temperature (°C)	TCC (mmol/g)	FCC (mmol/g)	FCC/TCC	DS
US—Fumaric	110	1.3	0.17	0.13	0.20
JP—Fumaric	110	1.5	0.22	0.14	0.24
US—Citric	130	2.6	0.47	0.18	0.45
JP—Citric	130	2.7	0.55	0.20	0.47

**Table 3:** Comparison of TCC values obtained by different reported methods.

Method	Substrate	TCC (mmol/g)	Ref.
Solid-state reaction (this work)	Cotton	4.0	This work
TEMPO	Cellulose	1.5	[11]
Chloroacetate	Eucalyptus kraft pulp	0.31	[24]
Chloroacetate	Cotton	0.74	[25]
Molten Oxalic acid	Cellulose pulp	3.3	[26]

## Data Availability

Not applicable.

## Supplementary Materials

The following are available online at https://www.mdpi.com/article/10.3390/membranes11070514/s1, Figure S1. FTIR spectra of cotton samples treated with oxalic acid at different times, Figure S2. FTIR spectra of cotton samples treated with fumaric acid at different times, Figure S3. FTIR spectra of cotton samples treated with citric acid at different times, Figure S4. Total carboxyl group content of samples treated with oxalic acid at 110 °C as a function of time, Figure S5. Free carboxyl content/total carboxyl content ratio of samples treated with oxalic acid at 110 °C as a function of time, Figure S6. Degree of substitution of samples treated with oxalic acid at 110 °C as a function of time, Figure S7. Total carboxyl group content of samples treated with fumaric acid at 110 °C as a function of time, Figure S8. Free carboxyl content/total carboxyl content ratio of samples treated with fumaric acid at 110 °C as a function of time, Figure S9. Degree of substitution of samples treated with fumaric acid at 110 °C as a function of time. Figure S10. Total carboxyl group content of samples treated with citric acid at 110 °C as a function of time, Figure S11. Free carboxyl content/total carboxyl content ratio of samples treated with citric acid at 110 °C as a function of time, Figure S12. Degree of substitution of samples treated with citric acid at 110 °C as a function of time, Figure S13. Total carboxyl group content of samples treated with fumaric acid for 45 min as a function of temperature, Figure S14. Free carboxyl content/total carboxyl content ratio of samples treated with fumaric acid for 45 min as a function of temperature, Figure S15. Degree of substitution of samples treated with fumaric acid for 45 min as a function of temperature, Figure S16. Total carboxyl group content of samples treated with citric acid for 45 min as a function of temperature, Figure S17. Free carboxyl content/total carboxyl content ratio of samples treated with citric acid for 45 min as a function of temperature, Figure S18. Degree of substitution of samples treated with citric acid for 45 min as a function of temperature, Figure S19. Methylene blue uptake isotherm on untreated cotton. Figure S20. Langmuir linearization of the methylene blue uptake isotherm on untreated cotton, Figure S21. Freundlich linearization of the methylene blue uptake isotherm on untreated cotton. Figure S22. Methylene blue uptake isotherm on oxalic acid-modified cotton. Figure S23. Langmuir linearization of the methylene blue uptake isotherm on oxalic acid-modified cotton, Figure S24. Freundlich linearization of the methylene blue uptake isotherm on oxalic acid-modified cotton, Figure S25. Methylene blue uptake isotherm on fumaric acid-modified cotton, Figure S26. Langmuir linearization of the methylene blue uptake isotherm on fumaric acid-modified cotton, Figure S27. Freundlich linearization of the methylene blue uptake isotherm on fumaric acid-modified cotton, Figure S28. Methylene blue uptake isotherm on citric acid-modified cotton, Figure S29. Langmuir linearization of the methylene blue uptake isotherm on citric acid-modified cotton. Figure S30. Freundlich linearization of the methylene blue uptake isotherm on citric acid-modified cotton, Figure S31. Methylene blue uptake pseudo first-order adsorption kinetics on oxalic acid-modified cotton, Figure S32. Methylene blue uptake pseudo second-order adsorption kinetics on oxalic acid-modified cotton, Figure S33. Methylene blue uptake intra-particle on oxalic acid-modified cotton, Figure S34. Methylene blue uptake pseudo first-order adsorption kinetics on fumaric acid-modified cotton, Figure S35. Methylene blue uptake pseudo second-order adsorption kinetics on fumaric acid-modified cotton, Figure S36. Methylene blue uptake intra-particle on fumaric acid-modified cotton, Figure S37. Methylene blue uptake pseudo first-order adsorption kinetics on citric acid-modified cotton, Figure S38. Methylene blue uptake pseudo second-order adsorption kinetics on citric acid-modified cotton, Figure S39. Methylene blue uptake intra-particle on citric acid-modified cotton.
