# Supplementary material for: A Solid-State Pathway towards the Tunable Carboxylation of Cellulosic Fabrics: Controlling the Surface’s Acidity
Source: Membranes (Basel). 2021 Jul 8;11(7):514. doi: 10.3390/membranes11070514 (PMC8303120; doi:10.3390/membranes11070514)
Supplement: Supplementary file 1 [file membranes-11-00514-s001.zip › membranes-1284655-supplementary.pdf]

membranes

MDPI

Supplementary Material

A Solid-State Pathway towards the Tunable Carboxylation of Cellulosic Fabrics: Controlling the Surface's Acidity

Eugenio H. Otal [1,*], Manuela L. Kim [1], Juan Hinestroza [4], Mutsumi Kimura [1,2,3*]

1 Department of Chemistry and Materials, Faculty of Textile Science and Technology, Shinshu University, Ueda Campus, 386-8567, Japan.
2 COI Aqua-Innovation Center, Shinshu University, Ueda Campus, 386-8567, Japan.
3 Research Initiative for Supra-Materials, Shinshu University, Ueda Campus, 386-8567, Japan.
4 Department of Fiber Science and Apparel Design, Cornell University, Ithaca, NY 14853, USA.
* Correspondence: eugenio_otal@shinshu-u.ac.jp, mkimura@shinshu-u.ac.jp

1. FTIR

1.1. Oxalic Acid

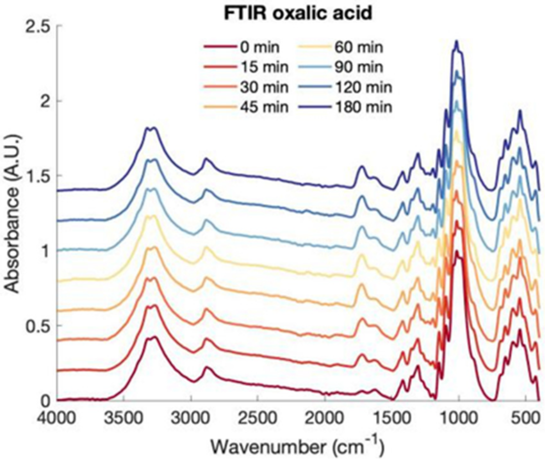

Figure S1. FTIR spectra of cotton samples treated with oxalic acid at different times.

1.2. Fumaric Acid

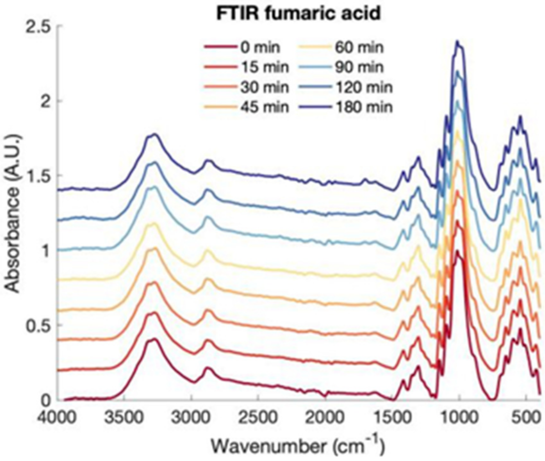

Figure S2. FTIR spectra of cotton samples treated with fumaric acid at different times.

1.3 Citric Acid

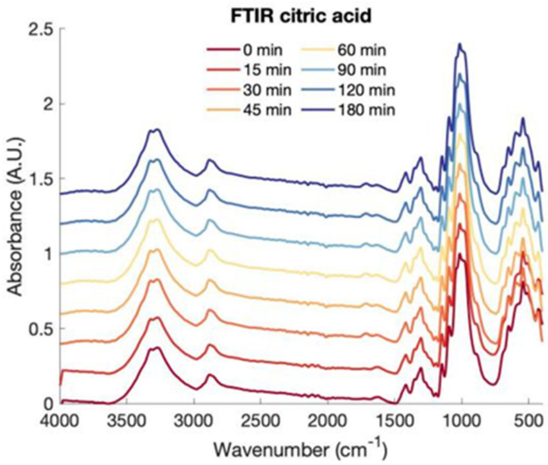

Figure S3. FTIR spectra of cotton samples treated with citric acid at different times.

2. Carboxyl Groups Titration

2.1. Time Optimization

Oxalic Acid—Total Carboxyl Content (TCC)

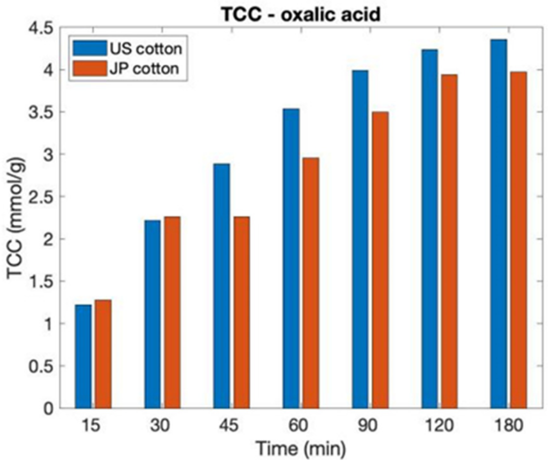

Figure S4. Total carboxyl group content of samples treated with oxalic acid at 110 °C as a function of time.

Free Carboxyl Content/Total Carboxyl Content Ratio

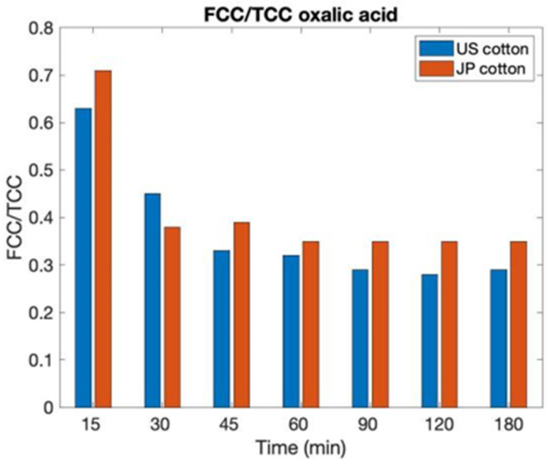

Figure S5. Free carboxyl content/total carboxyl content ratio of samples treated with oxalic acid at 110 °C as a function of time.

Degree of Substitution

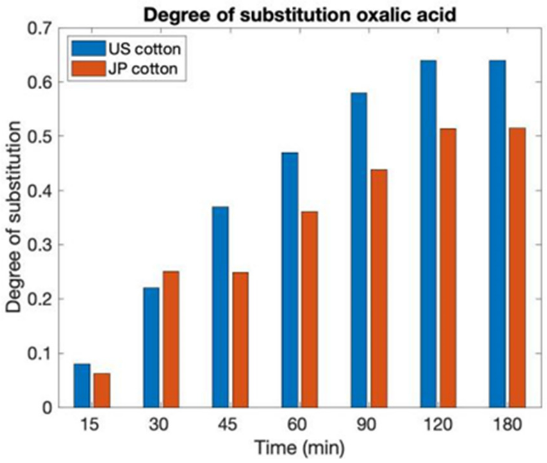

Figure S6. Degree of substitution of samples treated with oxalic acid at 110 °C as a function of time.

Fumaric Acid—Total Carboxyl Content (TCC)

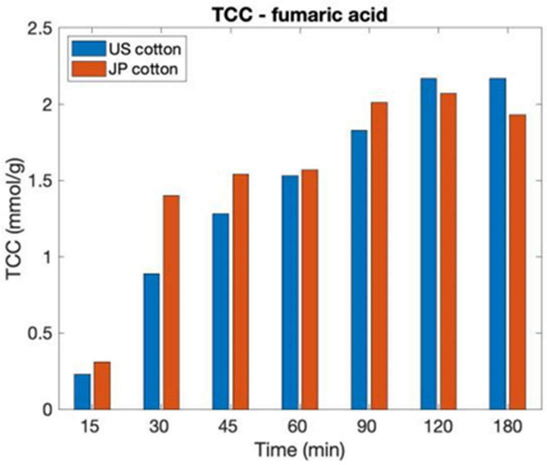

Figure S7. Total carboxyl group content of samples treated with fumaric acid at 110 °C as a function of time.

Free Carboxyl Content/Total Carboxyl Content Ratio

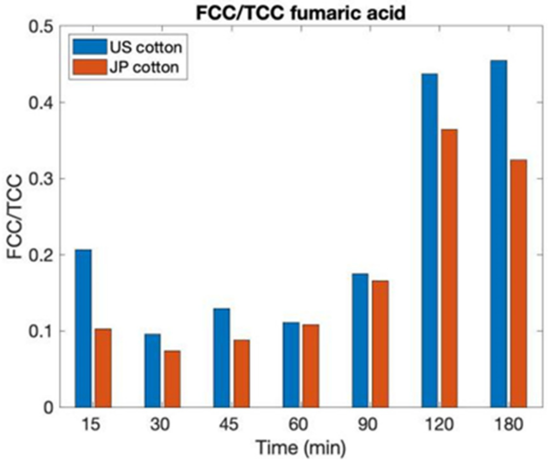

Figure S8. Free carboxyl content/total carboxyl content ratio of samples treated with fumaric acid at 110 °C as a function of time.

Degree of Substitution

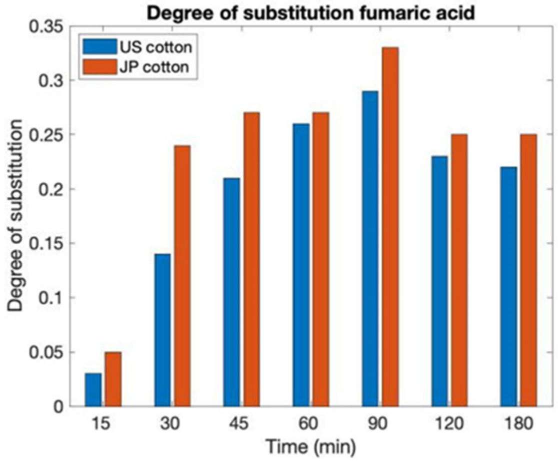

Figure S9. Degree of substitution of samples treated with fumaric acid at 110 °C as a function of time.

Citric Acid—Total Carboxyl Content (TCC)

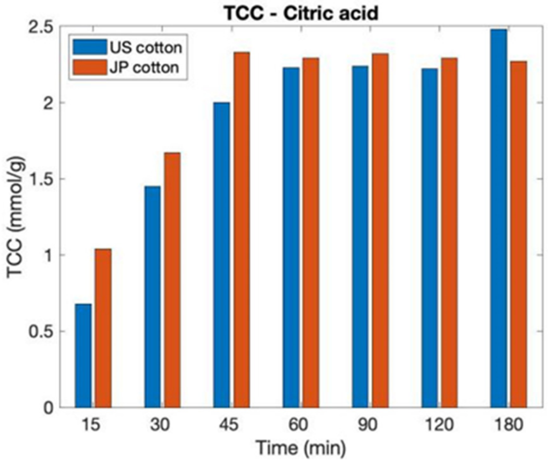

Figure S10. Total carboxyl group content of samples treated with citric acid at 110 °C as a function of time.

Free Carboxyl Content/Total Carboxyl Content Ratio

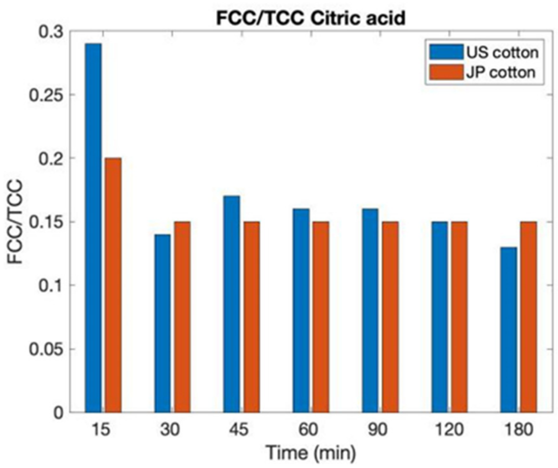

Figure S11. Free carboxyl content/total carboxyl content ratio of samples treated with citric acid at 110 °C as a function of time.

Degree of Substitution

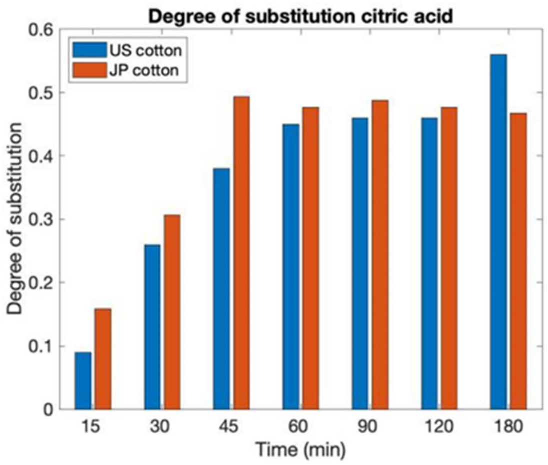

Figure S12. Degree of substitution of samples treated with citric acid at 110 °C as a function of time.

2.2. *Temperature Optimization*

Fumaric Acid—Total Carboxyl Content (TCC)

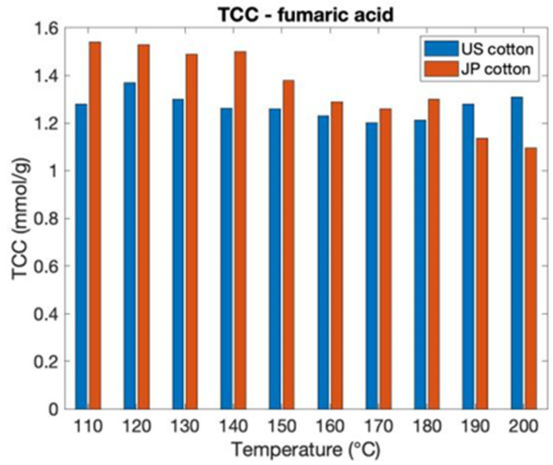

Figure 13. Total carboxyl group content of samples treated with fumaric acid for 45 min as a function of temperature.

Free Carboxyl Content/Total Carboxyl Content Ratio

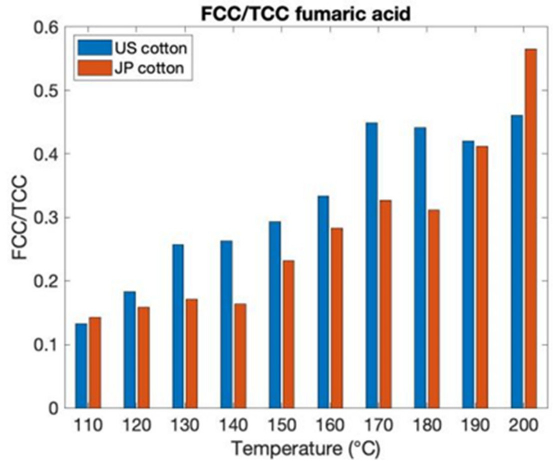

Figure S14. Free carboxyl content/total carboxyl content ratio of samples treated with fumaric acid for 45 min as a function of temperature.

Degree of Substitution

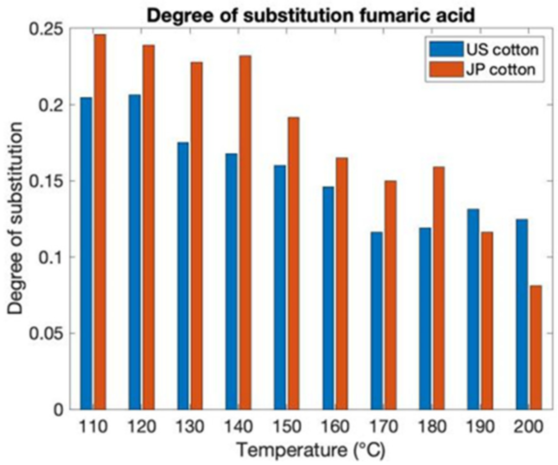

Figure S15. Degree of substitution of samples treated with fumaric acid for 45 min as a function of temperature.

Citric Acid—Total Carboxyl Content (TCC)

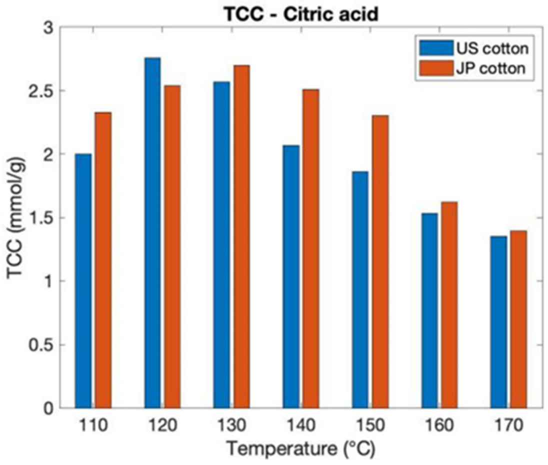

Figure S16. Total carboxyl group content of samples treated with citric acid for 45 min as a function of temperature.

Free Carboxyl Content/Total Carboxyl Content Ratio

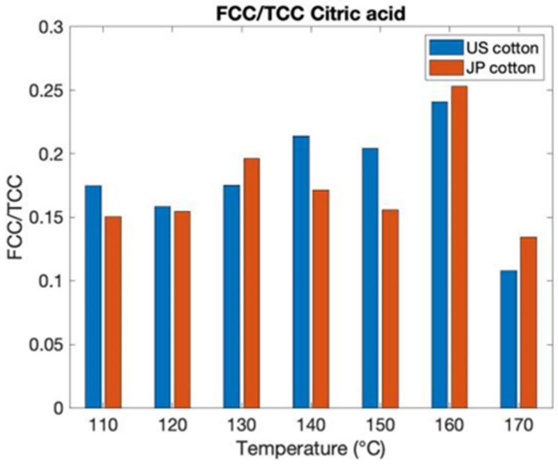

Figure S17. Free carboxyl content/total carboxyl content ratio of samples treated with citric acid for 45 min as a function of temperature.

Degree of Substitution

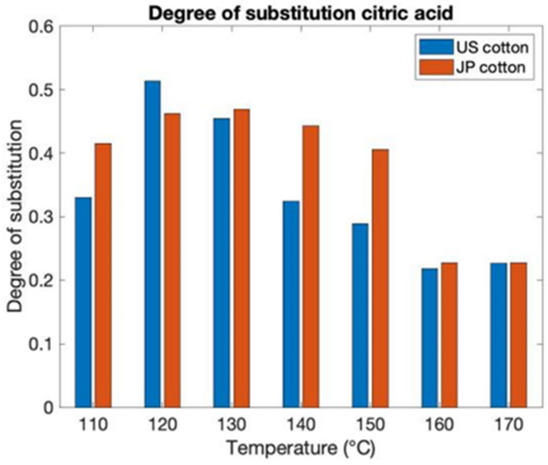

Figure S18. Degree of substitution of samples treated with citric acid for 45 min as a function of temperature.

1.3. *Methylene Blue Adsorption Isotherms*

Untreated Cotton

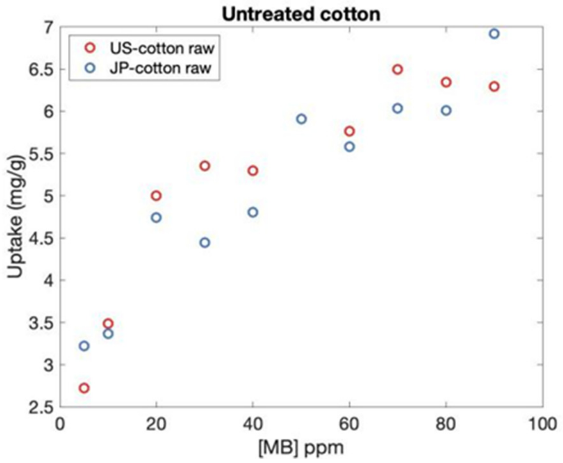

Figure S19. Methylene blue uptake isotherm on untreated cotton.

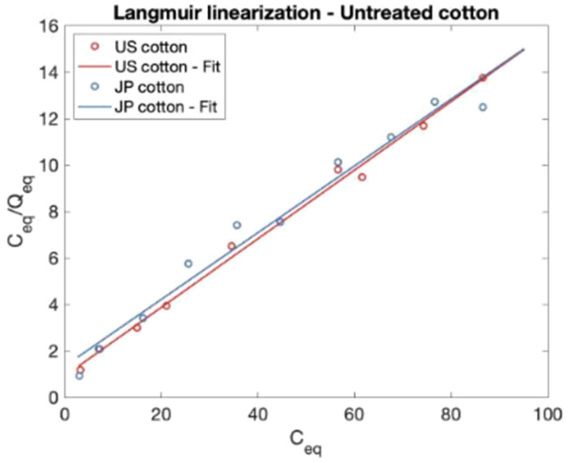

Figure S20. Langmuir linearization of the methylene blue uptake isotherm on untreated cotton.

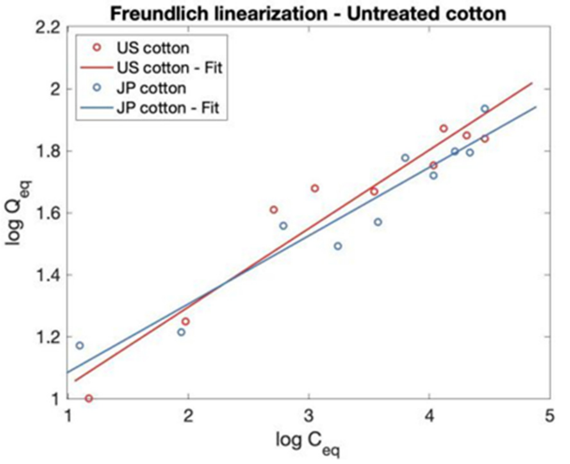

Figure S21. Freundlich linearization of the methylene blue uptake isotherm on untreated cotton.

Oxalic Acid

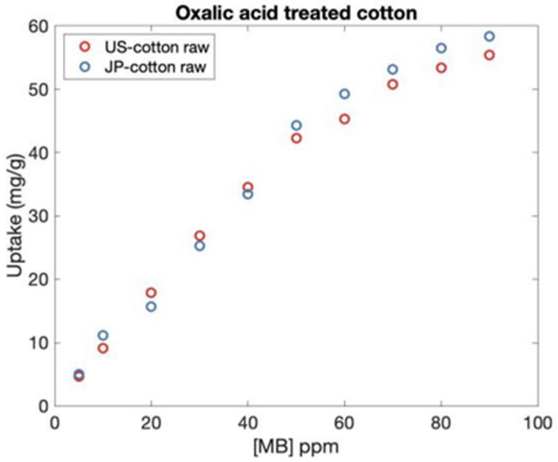

Figure 22. Methylene blue uptake isotherm on oxalic acid-modified cotton.

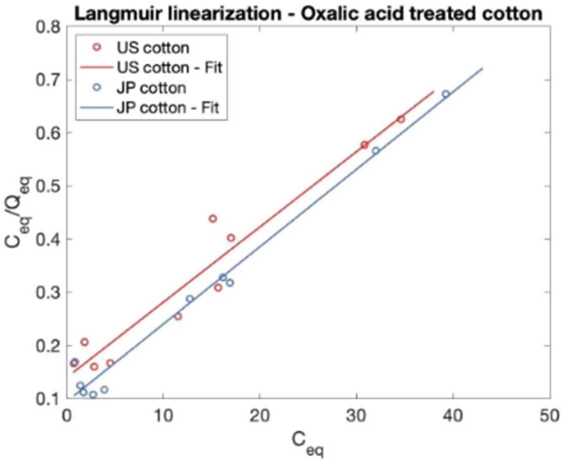

Figure S23. Langmuir linearization of the methylene blue uptake isotherm on oxalic acid-modified cotton.

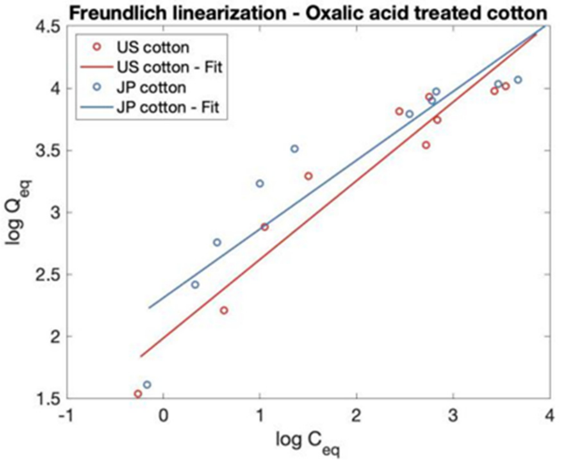

Figure S24. Freundlich linearization of the methylene blue uptake isotherm on oxalic acid-modified cotton.

Fumaric Acid

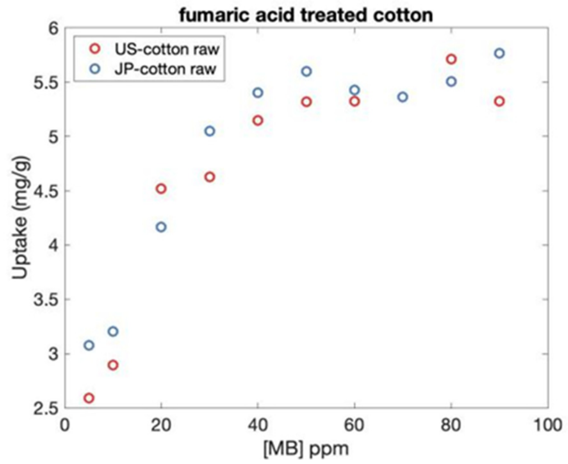

Figure S25. Methylene blue uptake isotherm on fumaric acid-modified cotton.

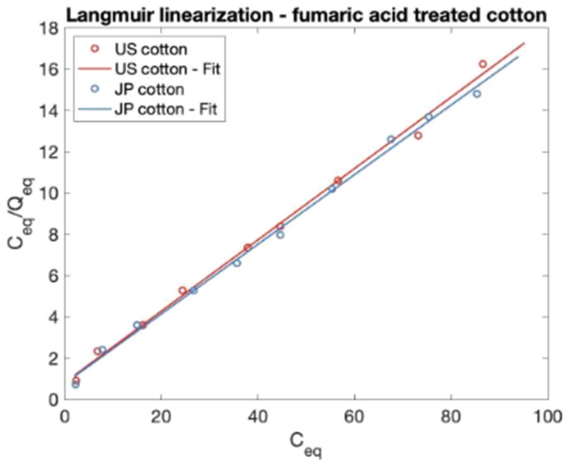

Figure S26. Langmuir linearization of the methylene blue uptake isotherm on fumaric acid-modified cotton.

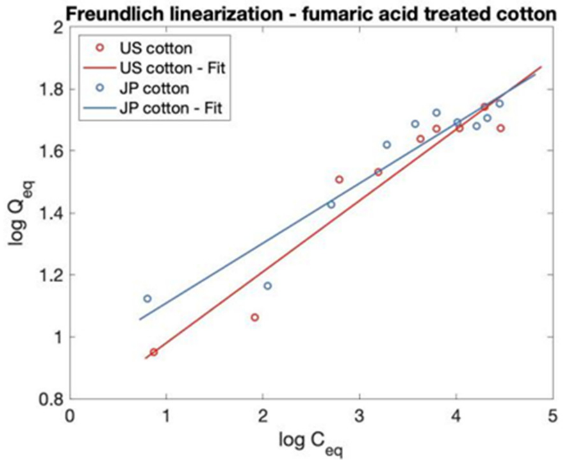

Figure S27. Freundlich linearization of the methylene blue uptake isotherm on fumaric acid-modified cotton.

Citric Acid

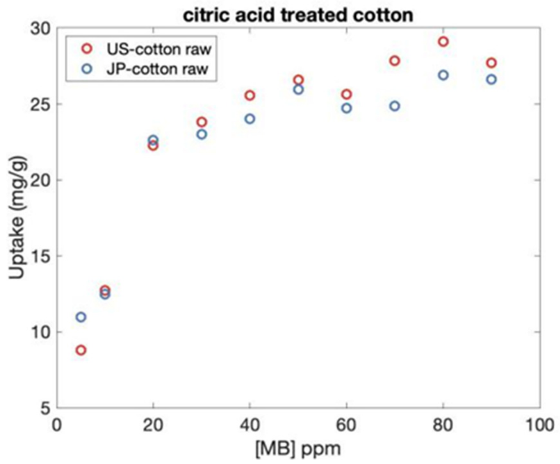

Figure S28. Methylene blue uptake isotherm on citric acid-modified cotton.

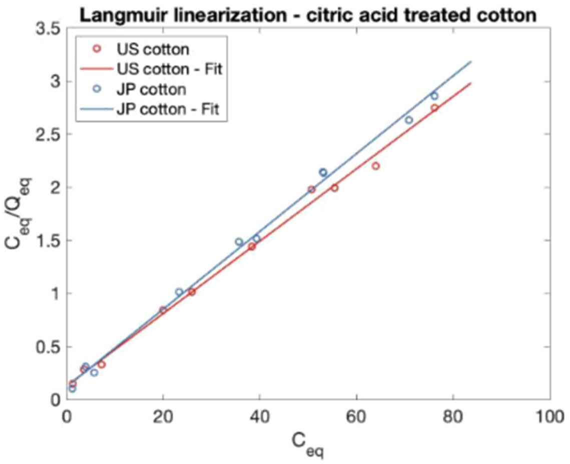

Figure S29. Langmuir linearization of the methylene blue uptake isotherm on citric acid-modified cotton.

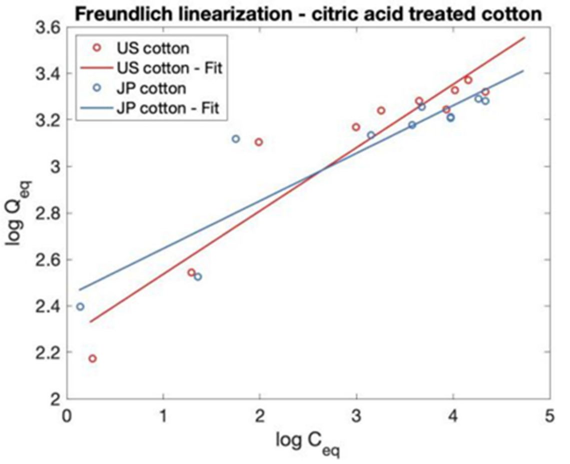

Figure S30. Freundlich linearization of the methylene blue uptake isotherm on citric acid-modified cotton.

1.4. Methylene Blue Adsorption Kinetics

Oxalic Acid-Treated Cotton

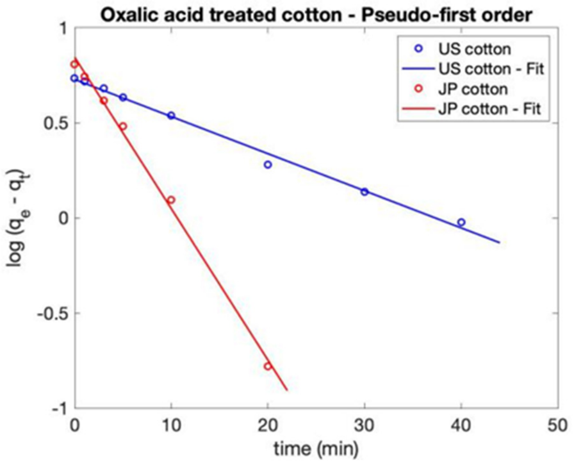

Figure S31. Methylene blue uptake pseudo first-order adsorption kinetics on oxalic acid-modified cotton.

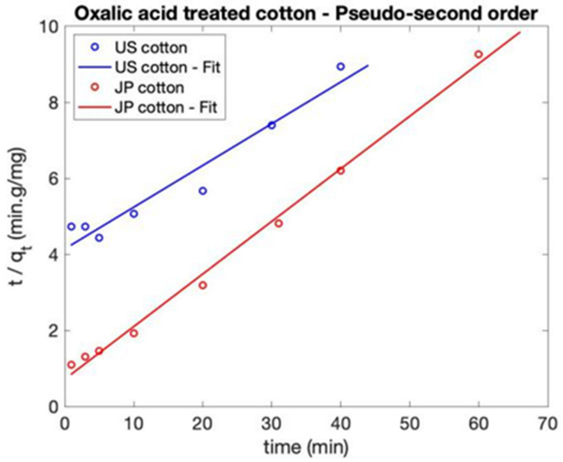

Figure S32. Methylene blue uptake pseudo second-order adsorption kinetics on oxalic acid-modified cotton.

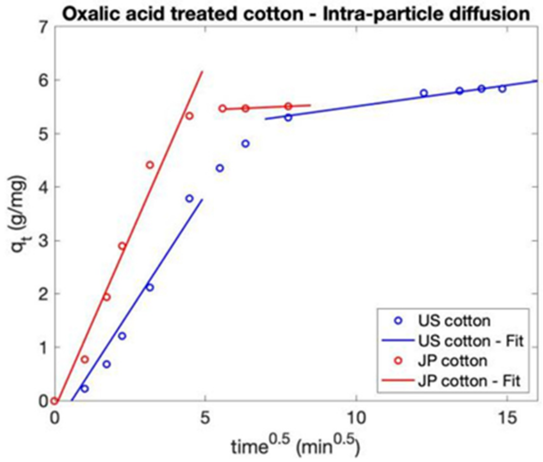

Figure S33. Methylene blue uptake intra-particle on oxalic acid-modified cotton.

Fumaric Acid-Treated Cotton

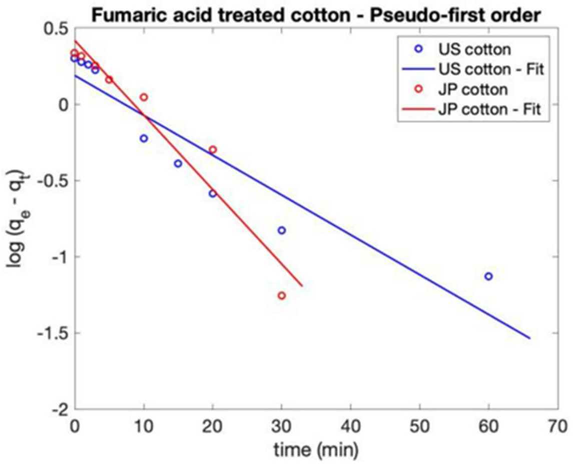

Figure S34. Methylene blue uptake pseudo first-order adsorption kinetics on fumaric acid-modified cotton.

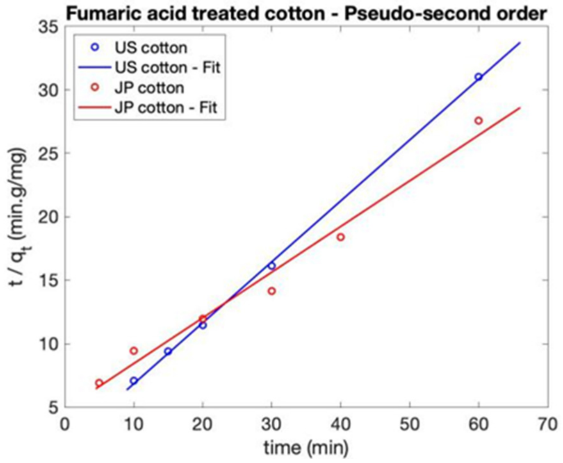

Figure S35. Methylene blue uptake pseudo second-order adsorption kinetics on fumaric acid-modified cotton.

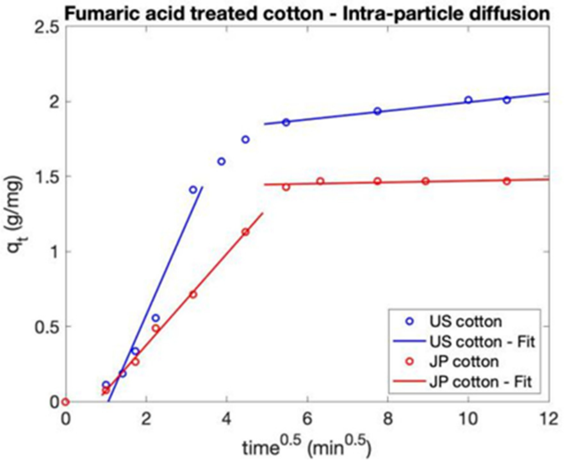

Figure S36. Methylene blue uptake intra-particle on fumaric acid-modified cotton.

Citric Acid-Treated Cotton

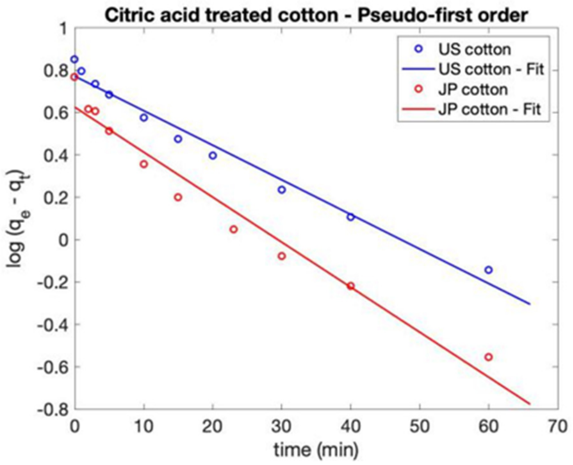

Figure S37. Methylene blue uptake pseudo first-order adsorption kinetics on citric acid-modified cotton.

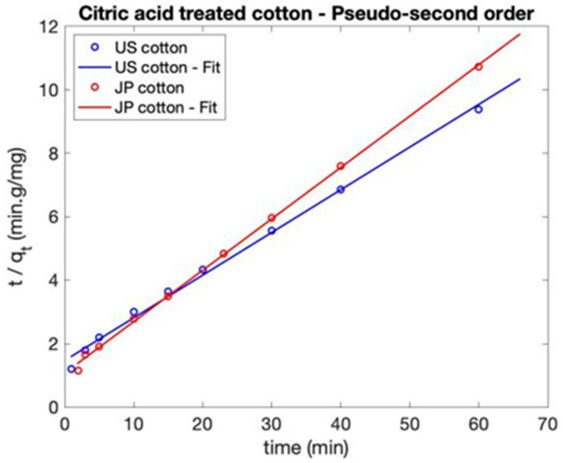

Figure S38. Methylene blue uptake pseudo second-order adsorption kinetics on citric acid-modified cotton.

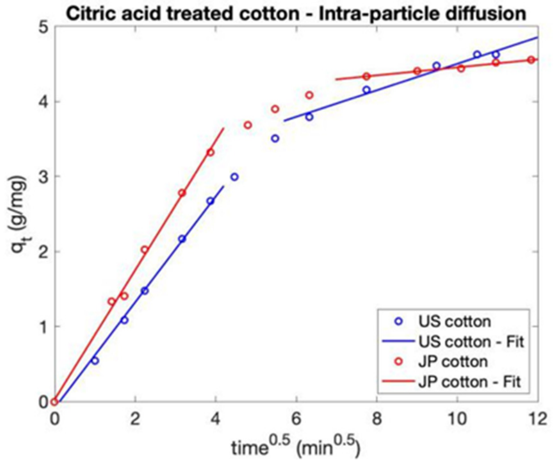

Figure S39. Methylene blue uptake intra-particle on citric acid-modified cotton.
